# A breast cancer classification and immune landscape analysis based on cancer stem-cell-related risk panel

**DOI:** 10.1038/s41698-023-00482-w

**Published:** 2023-12-08

**Authors:** Haihong Hu, Mingxiang Zou, Hongjuan Hu, Zecheng Hu, Lingxiang Jiang, David Escobar, Hongxia Zhu, Wendi Zhan, Ting Yan, Taolan Zhang

**Affiliations:** 1 Department of Pharmacy, The First Affiliated Hospital, Hengyang Medical School, University of South China, Hengyang, Hunan 421000 China; 2 School of Pharmacy, Hengyang Medical College, University of South China, 28 Western Changsheng Road, Hengyang, Hunan 421001 China; 3 Phase I clinical trial center, The First Affiliated Hospital, Hengyang Medical School, University of South China, Hengyang, Hunan 421000 China; 4 Department of Spine Surgery, The First Affiliated Hospital, Hengyang Medical School, University of South China, Hengyang, Hunan 421000 China; 5 Department of Public Health Service, The First Affiliated Hospital, Hengyang Medical School, University of South China, Hengyang, Hunan 421000 China; 6 Department of Nursing, The First Affiliated Hospital, Hengyang Medical School, University of South China, Hengyang, Hunan 421000 China; 7 Department of Breast and Thyroid Surgery, The First Affiliated Hospital, Hengyang Medical School, University of South China, Hengyang, Hunan 421000 China; 8 Department of Radiation Oncology, Melvin and Bren Simon Comprehensive Cancer Center, Indiana University School of Medicine, Indianapolis, IN 46202 USA; 9 Department of Cancer Biology, College of Medicine & Life Sciences, University of Toledo, Toledo, OH 43614 USA

**Keywords:** Prognostic markers, Cancer genomics

## Abstract

This study sought to identify molecular subtypes of breast cancer (BC) and develop a breast cancer stem cells (BCSCs)-related gene risk score for predicting prognosis and assessing the potential for immunotherapy. Unsupervised clustering based on prognostic BCSC genes was used to determine BC molecular subtypes. Core genes of BC subtypes identified by non-negative matrix factorization algorithm (NMF) were screened using weighted gene co-expression network analysis (WGCNA). A risk model based on prognostic BCSC genes was constructed using machine learning as well as LASSO regression and multivariate Cox regression. The tumor microenvironment and immune infiltration were analyzed using ESTIMATE and CIBERSORT, respectively. A CD79A^+^CD24^-^PANCK^+^-BCSC subpopulation was identified and its spatial relationship with microenvironmental immune response state was evaluated by multiplexed quantitative immunofluorescence (QIF) and TissueFAXS Cytometry. We identified two distinct molecular subtypes, with Cluster 1 displaying better prognosis and enhanced immune response. The constructed risk model involving ten BCSC genes could effectively stratify patients into subgroups with different survival, immune cell abundance, and response to immunotherapy. In subsequent QIF validation involving 267 patients, we demonstrated the existence of CD79A^+^CD24^-^PANCK^+^-BCSC in BC tissues and revealed that this BCSC subtype located close to exhausted CD8^+^FOXP3^+^ T cells. Furthermore, both the densities of CD79A^+^CD24^-^PANCK^+^-BCSCs and CD8^+^FOXP3^+^T cells were positively correlated with poor survival. These findings highlight the importance of BCSCs in prognosis and reshaping the immune microenvironment, which may provide an option to improve outcomes for patients.

## Introduction

Breast cancer (BC) has become the most common cancer in women with a highly heterogeneous malignancy occurring in breast tissue. According to statistics released by the International Agency for Research on Cancer (IARC) of the World Health Organization (WHO), there were approximately 2.3 million new cases of breast cancer worldwide in 2020 and the mortality rate was highly reached to 15.5% in women^1^. The heterogeneity nature of BC poses challenges for precise treatment, including surgery, chemotherapy, radiotherapy, and emerging immunotherapy, leading to clinical issues such as recurrence, metastasis, and drug resistance^2,3^. Current clinical, pathological, and hormonal staging systems fall short of providing a comprehensive understanding of BC heterogeneity. Therefore, exploration and identification of molecular classifications are crucial to address this complexity.

Mounting evidence suggests that breast cancer stem cells (BCSCs), possessing potent tumorigenic properties, self-renewal capabilities, and multi-differentiation potential, may underlie the origin of diverse tumor subsets within BC^4–6^. In addition, BCSCs contribute significantly to metastasis, recurrence, and resistance to conventional therapies, including surgery, radiotherapy, chemotherapy, and targeted therapy^7,8^. Previous studies have revealed that BCSCs could interact with tumor-infiltrating immune cells such as CD8 T cells, influencing the tumor microenvironment and immunotherapy outcomes^9^. Accordingly, analysis of the characteristics related to cancer stem cells holds promise for precise breast cancer typing and provides insights into the immune landscape, potentially enhancing the diagnosis and treatment of BC patients.

In this study, we employed unsupervised clustering analysis based on the expression of cancer stem cell-related genes to identify two distinct molecular subtypes of BC. Subsequent investigations revealed that Cluster 1 exhibited better survival outcomes, likely attributable to its enhanced immune response. Weighted gene co-expression network analysis (WGCNA) and differential analysis were employed to identify core genes within Cluster 1 and explore the associated biological processes, indicating significant enrichment in T-cell and B-cell activation signaling pathways. Using machine learning, we constructed breast cancer stem-cell-related risk scores (BCSCRS) based on the prognosis-related stem cell genes. We then analyzed the molecular characterization and immune landscape of BCSCRS, validating their accuracy and applicability for predicting BC prognosis. Furthermore, our study revealed a stem cell population named CD79A^+^CD24^-^PANCK^+^-BCSCs subpopulation with poor prognosis. The strong interaction between CD79A^+^CD24^-^PANCK^+^-BCSCs subpopulation and exhausted CD8^+^ T cells with FOXP3^+^, suggesting that CD79A^+^CD24^-^PANCK^+^-BCSCs subpopulation may play an important role in the immunosuppressive microenvironment by exhausting CD8^+^T cells.

In conclusion, we revealed breast cancer subtypes based on BCSCs-related genes and developed the BCSCs-related risk panel for predicting prognosis and analyzing immune landscape. Furthermore, the complex interplay identified between the CD79A^+^CD24^-^PANCK^+^-BCSCs subpopulation and exhausted CD8^+^ T cells not only offered an avenue for improving prognosis in breast cancer but also emphasized the importance of breast cancer stem cells in the immunosuppressive microenvironment.

## Results

### Identification of two breast cancer stem-cell-related subtypes and their immune characteristics

**Fig. 1:**
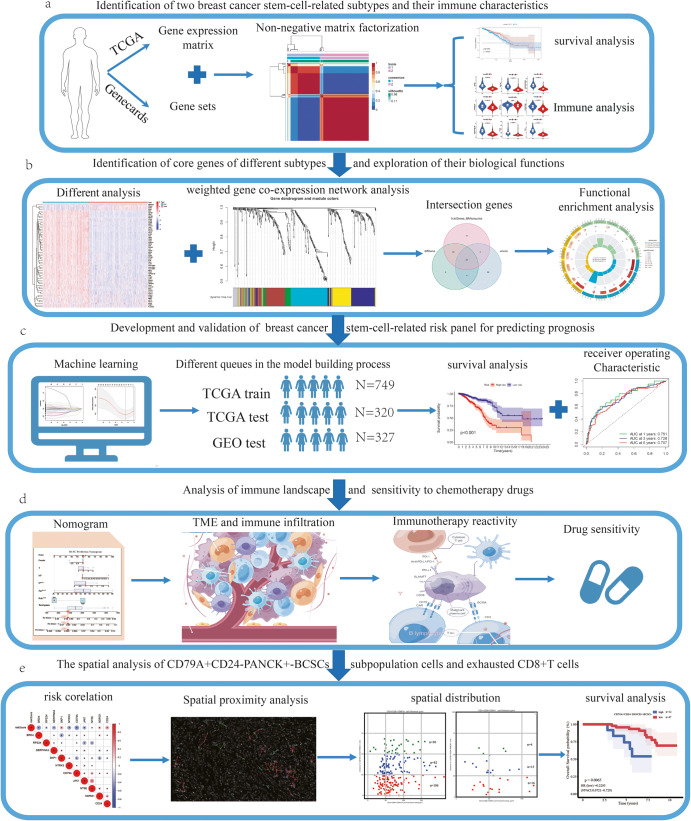
The flow chart for this article. **a** Identification of different molecular subtypes of breast cancer. **b** Identification of core genes of different subtypes and exploration of their biological functions. **c** Construction and validation of prognostic model. **d** Evaluation of the immunotherapy response and sensitivity of chemotherapy drugs. **e** Spatial proximity analysis of CD79A^+^CD24^-^PANCK^+^-BCSCs subpopulation in the tumor microenvironment.

**Fig. 2:**
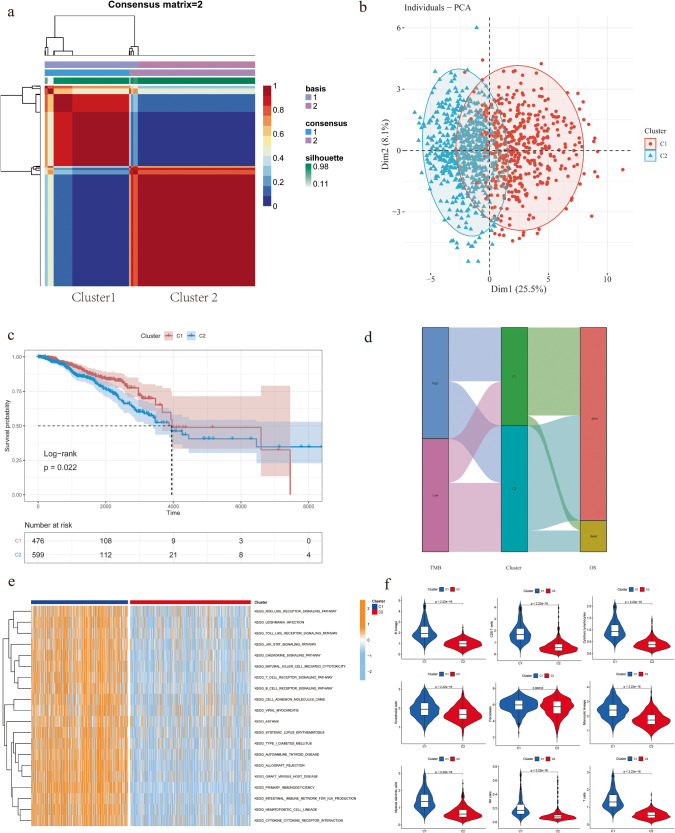
Breast cancer stem-cell-related subtypes and their characteristics. **a** Unsupervised clustering algorithm identified two distinct subtypes in the TCGA breast cancer cohort. **b** Principal component analysis of breast cancer stem-cell-related subtypes in the TCGA cohort (“Dim1” signifies the primary component that maximizes the variance and captures the most substantial differences between the samples. “Dim2” corresponds to the second most prominent component). **c** Kaplan–Meier curves of various breast cancer stem-cell-related subtypes in the TCGA cohort. **d** Alluvial diagram shows changes in breast cancer stem-cell-related subtypes, TMB (tumor mutation burden), and OS (overall survival). **e** GSVA analyzed the biological pathways of two breast cancer stem-cell-related subtypes. Red denotes the biological processes that are activated, while blue denotes the biological processes that are inhibited. **f** Immune infiltration analysis between two breast cancer stem-cell-related subtypes.

The flow chart depicting the methodology for this study is presented in Fig. 1. To perform patient clustering in breast cancer, we employed the NMF algorithm, an unsupervised machine learning method. Through this analysis of BCSC-related gene expression in the TCGA cohort, we identified two distinct subtypes. Cluster 1 comprised 476 cases, while Cluster 2 included 599 cases (Fig. 2a). The robustness of clustering results was performed principal component analysis (PCA), which further confirmed the separation of the two clusters, even in the presence of some overlapping data points (Fig. 2b). Subsequent survival analysis showed that patients in Cluster 1 exhibited a higher overall survival (OS) rate compared to those in Cluster 2 (Fig. 2c). Additionally, Cluster 1 had higher TMB values and higher mutation frequency (Supplementary Fig. 1). Interestingly, the proportion of higher TMB was notably more in Cluster 1 than in Cluster 2 (Fig. 2d), suggesting a potential association between BCSCs-related subtypes and TMB. This observation could have significant implications for the efficacy of immunotherapy, as higher TMB is known to correlate with a heightened response to immunotherapy^10^. To gain insights into the biological differences between two subtypes, gene set variation analysis (GSVA) was conducted, revealing significantly distinct functional regulation modes (Fig. 2e). Notably, Cluster 1 exhibited significant enrichment in immune-related signaling pathways, including the T-cell receptor signaling pathway, B-cell receptor signaling pathway, and primary immunodeficiency. Furthermore, we also assessed the differences in the TME between the two subtypes by calculating TME scores including ESTIMATE score, Immune score, Stromal score, and tumor purity, using the expression matrix from TCGA. The scores of samples in Cluster 1 were significantly higher than Cluster 2, except for tumor purity (Supplementary Fig. 2). Infiltration analysis using the MCPcounter package showed that Cluster 1 was associated with abundant infiltration of B lineage, CD8 T-cell, cytotoxic lymphocytes, myeloid dendritic cells, NK (natural killer) cells, and T cells (Fig. 2f), indicating a closer association with immune activity. Taken together, these findings suggest the existence of distinct subtypes of breast cancer characterized by variations in immune-related signaling pathways, TME, and survival outcomes.

### Recognition of core modules and genes in BCSC-related Cluster 1 and Cluster 2

**Fig. 3:**
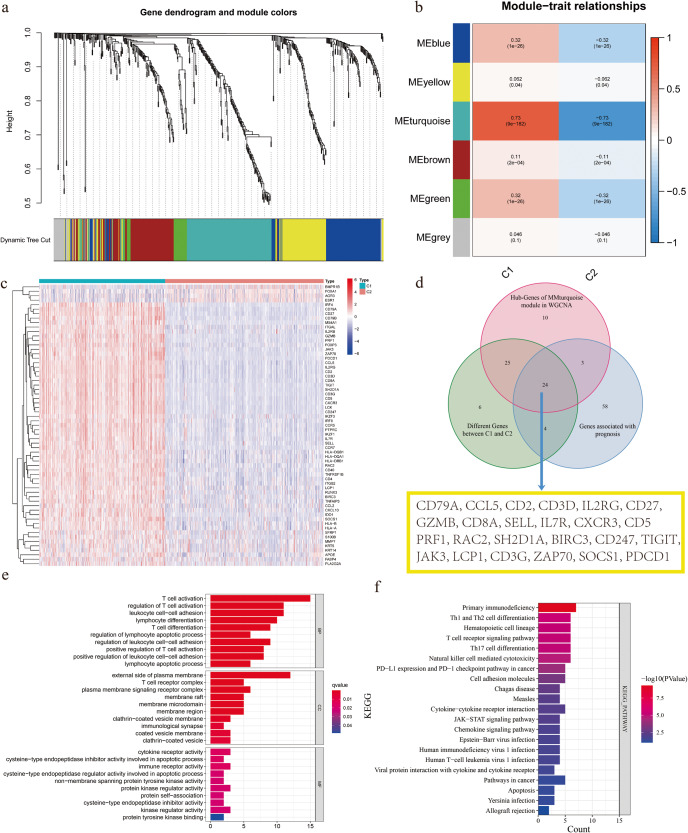
Identification and functional verification of critical gene modules. **a** WGCNA analysis based on breast cancer stem cells-related gene expression data identified gene modules with high covariance. **b** Heat map of module-trait relationships. **c** Differential expression analysis of genes in Cluster 1 (C1) and Cluster 2 (C2). **d** Venn diagram shows the intersection of the differential gene of Cluster 1 (C1) and Cluster 2 (C2), the hub genes in the core module and prognosis genes. **e** GO (Gene Ontology) enrichment analysis of intersection genes. **f** KEGG (Kyoto Encyclopedia of Genes and Genomes) enrichment analysis of intersection genes.

To identify critical gene modules within the BCSC-related clusters, we performed WGCNA on the expression matrix from the TCGA cohort, resulting in six co-expression modules (Fig. 3a). Among these modules, the ME turquoise module exhibited the strongest correlation with Cluster 1, as evident from the heat map depicting the module-trait relationship, while Cluster 2 showed a weaker correlation (Fig. 3b). Furthermore, the ME turquoise module demonstrated the highest values for the important index and correlation coefficient. From this module, we identified 62 hub genes for further investigation. To gain deeper insights, we performed differential analysis using the limma package to identify genes highly expressed in Cluster 1. As a result, we obtained 63 differential genes, with only four genes highly expressed in Cluster 2, while 59 genes were upregulated in Cluster 1 (Fig. 3c). Using these core and differential genes, we performed biological function verification and identified 24 survival-related genes for GO and KEGG enrichment analysis (Fig. 3d). The GO analysis demonstrated significant enrichment of the identified gene sets in the activation and differentiation of T cells (Fig. 3e). Furthermore, the KEGG results indicated their involvement in processes related to primary immunodeficiency, Th1 and Th2 cell differentiation, and T-cell receptor signaling pathways (Fig. 3f). These findings strongly suggest a pronounced correlation between BCSCs-related genes and immune activity, particularly in biological processes related to T cells.

### Development and validation of breast cancer stem-cell-related risk panel for predicting prognosis

**Fig. 4:**
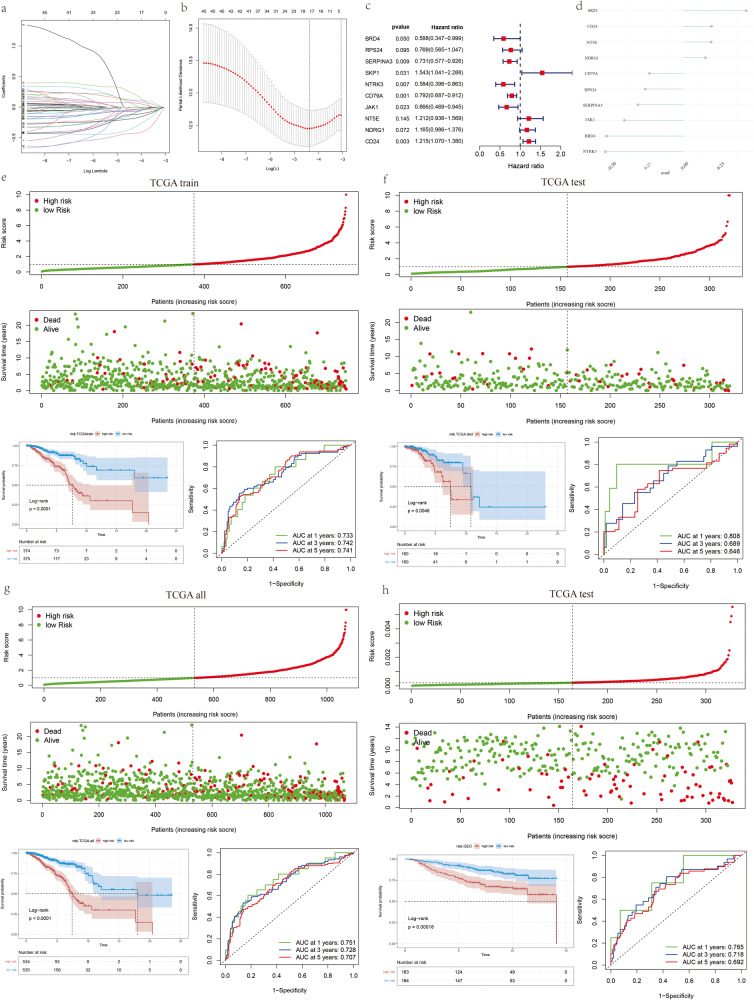
Development and validation of a BCSC-related prognostic signature. **a**, **b** Least absolute shrinkage and selection operator (LASSO) further screen for genes associated with prognosis. **c** The forest plot displays the results of survival analysis for different genes. Each horizontal line represents a gene, with the line’s length indicating its Hazard Ratio (HR), and the arrows representing the 95% confidence interval for the HR. HR values greater than 1 indicate an increased risk, while HR values less than 1 indicate a decreased risk. **d** Coefficient of the prognostic model was used to calculate the risk score. Survival scatter plot, Kaplan–Meier analyses, time-dependent ROC (receiver operating characteristic) curve analyses at 1, 3, and 5 years in the TCGA training cohort (**e**), TCGA test cohort (**f**), TCGA all cohort (**g**), GSE20685 test cohort (**h**).

In previous investigation, we successfully identified two subtypes of breast cancer that were associated with breast cancer stem cells and thoroughly explored the biological functions of their core genes. Our subsequent objective was to develop a BCSCs-related model for predicting the prognosis of breast cancer patients. To achieve this, a cohort of 1069 breast cancer patients, with information on survival state and overall survival time, was obtained from the TCGA database, and was randomly split into a training cohort and an internal validation cohort at a ratio of 7:3. From the training cohort of 749 patients, we identified 45 BCSCs-related genes that were associated with survival. Next, we performed LASSO regression analysis, which led to the selection of 17 BCSC-related genes for further multivariate Cox regression (Fig. 4a, b). With these findings, we then constructed a prognosis model based on multivariate Cox regression analysis, which revealed ten genes forming the BCSC-related risk pane (Fig. 4c). The BCSCRS was calculated using the following formula, as depicted in Fig. 4d and Table 1: BCSCRS= (−0.53045 × BRD4) + ( − 0.26259 × RPS24) + ( − 0.31334×SERPINA3) + (0.434039 × SKP1) + ( − 0.53742×NTRK3) + ( − 0.23344 × CD79A) + ( − 0.40628 × JAK1) + (0.192005×NT5E) + (0.152866 × NDRG1) + (0.194872 × CD24). All patients were divided into high- and low-risk groups based on the median BCSCRS. Notably, in the training cohort (*N* = 749), the low-risk group (*N* = 374) exhibited significantly better overall survival compared to the high-risk group (*N* = 375). To assess the predictive performance of BCSCRS, time-dependent ROC curve analysis was conducted, yielding encouraging results with AUCs of 0.733 (1-year), 0.742 (3-year), and 0.741 (5-year) (Fig. 4e). These favorable outcomes were consistently observed in the TCGA test cohort, with AUCs of 0.808, 0.689, and 0.646 at 1-year, 3-year, and 5-year (Fig. 4f). Furthermore, these results were confirmed in the entire TCGA cohort and the GSE20685 cohort, demonstrating the high accuracy of BCSCRS in predicting survival (Fig. 4g, h).

**Table 1:** Coefficients of the 10 prognostic molecules in the Cox regression model.

id	coefficient	HR	HR.95 L	HR.95H	*p*-value
BRD4	−0.53045	0.588341	0.346529	0.998894	0.049523
RPS24	−0.26259	0.769056	0.564995	1.046818	0.095092
SERPINA3	−0.31334	0.731003	0.577071	0.925996	0.009397
SKP1	0.434039	1.543479	1.041318	2.287802	0.030649
NTRK3	−0.53742	0.584253	0.395529	0.863025	0.006932
CD79A	−0.23344	0.791808	0.687276	0.912239	0.001231
JAK1	−0.40628	0.666123	0.469321	0.94545	0.022971
NT5E	0.192005	1.211677	0.935775	1.568924	0.14527
NDRG1	0.152866	1.165169	0.986407	1.376328	0.072034
CD24	0.194872	1.215156	1.070033	1.379961	0.002672

### BCSCRS can serve as an independent prognostic factor

**Fig. 5:**
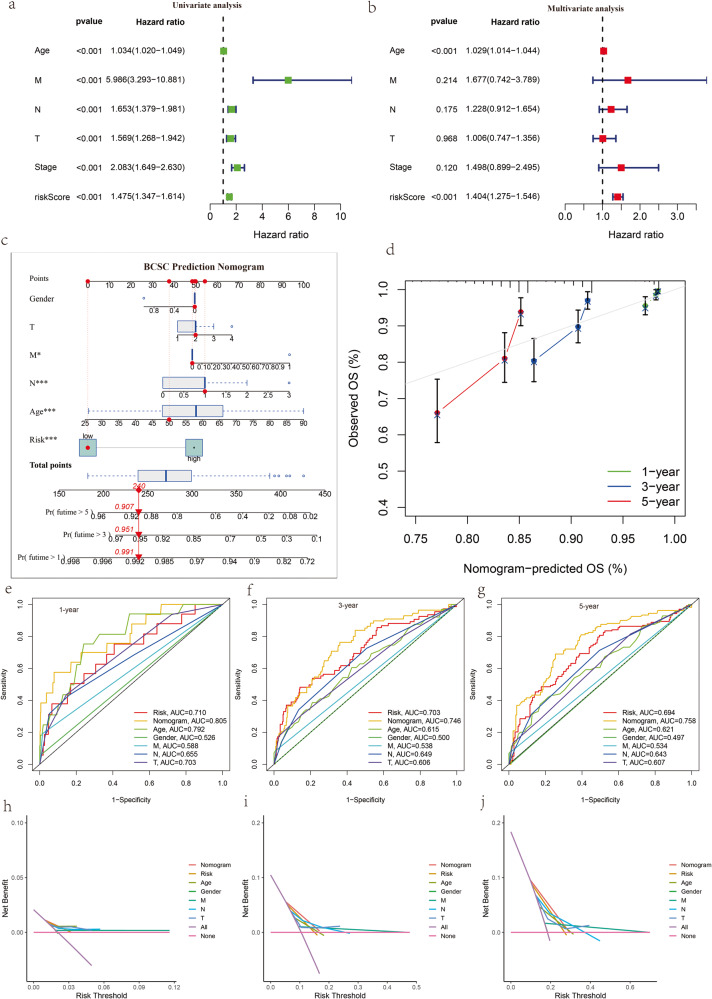
Development and validation of a prognostic nomogram. **a** Forest plot of univariate Cox regression analysis. **b** Forest plot of multivariate Cox regression analysis. **c** Nomogram predicting the probability of 1-, 3-, and 5-year survival for breast cancer patients based on risk score and clinical factors. **d** Calibration curves for the nomogram. **e**–**g** Receiver operating characteristic curves at 1, 3, and 5 years of the nomogram, BCSCRS, and clinical factors. **h**–**j** Decision curve analysis (DCA) of nomogram, BCSCRS, and clinical factors at 1, 3, and 5 years.

To investigate the potential independence of the BCSCRS as a prognostic factor, we performed both univariate and multivariate Cox regression analysis (Table 2). The results significantly indicated correlation between the risk score, age, stage, and TNM stage with the prognosis of breast cancer patients (Fig. 5a, *p* < 0.001). Furthermore, the multivariate Cox regression analysis showed that both the risk score and age could serve as independent prognostic factors for breast cancer patients (Fig. 5b, *p* < 0.001). To explore the potential associations between BCSCRS and various clinical variables, we conducted Wilcoxon and Kruskal–Wallis tests. Our analysis revealed that BCSCRS increased with tumor stage in the TCGA cohort, displaying significant differences between stages (Supplementary Fig. 3a). Notably, the risk score of T and N stages showed an upward trend, with significant distinctions between each group, while the opposite was found for N3 stages. Furthermore, BCSCRS was substantially higher in patients with advanced M stage and those over 65 years old. However, there was no statistically significant difference in risk score between various genders, likely due to the considerable difference in the number of cases. Similar results were obtained in the in the GSE20685 cohort, where the risk score was significantly higher in the advanced TNM stage (Supplementary Fig. 3b). These findings highlight the substantial variation of BCSCRS among different clinical variable groups, with higher risk scores indicating poorer pathological status in breast cancer patients. To incorporate the clinical factors related to survival, we constructed a nomogram as a quantitative method to predict the survival rate of breast cancer patients (Fig. 5c). The overall score of each patient was calculated by combining the BCSCRS and clinical variables, including gender, TNM stage, and age. Patients with lower total points were associated with a higher probability of survival. The accuracy of the nomogram was assessed by calibration curves (Fig. 5d) and the area under the ROC curve. The nomogram demonstrated improved predictive accuracy compared to other clinical features and the original risk score. The 1-year, 3-year, and 5-year AUCs of the nomogram in the TCGA cohort were 0.805, 0.746, and 0.758, respectively (Fig. 5e–g). Moreover, the results of DCA confirmed the better prediction accuracy of the nomogram compared to other prediction indexes (Fig. 5h–j).

**Table 2:** Univariate Cox regression and multivariate Cox regression of risk score and Clinical features.

	Univariate Cox regression		Multivariate Cox regression	
	HR	*p*-value	HR	*p-*value
Age^*^	1.034190404	2.88E-06	1.02914032	0.000121193
M	5.986194821	4.38E-09	1.677082391	0.213669472
N	1.652706164	5.63E-08	1.228363384	0.175110837
T	1.569486311	3.34E-05	1.006175755	0.967753232
Stage	2.082913144	7.17E-10	1.498060951	0.120448476
Risk score^*^	1.474573151	3.69E-17	1.403825677	5.01E-12

^*^Independent prognostic factors.

### Benefits of BCSCRS in comparison with other breast cancer prognostic signatures

**Fig. 6:**
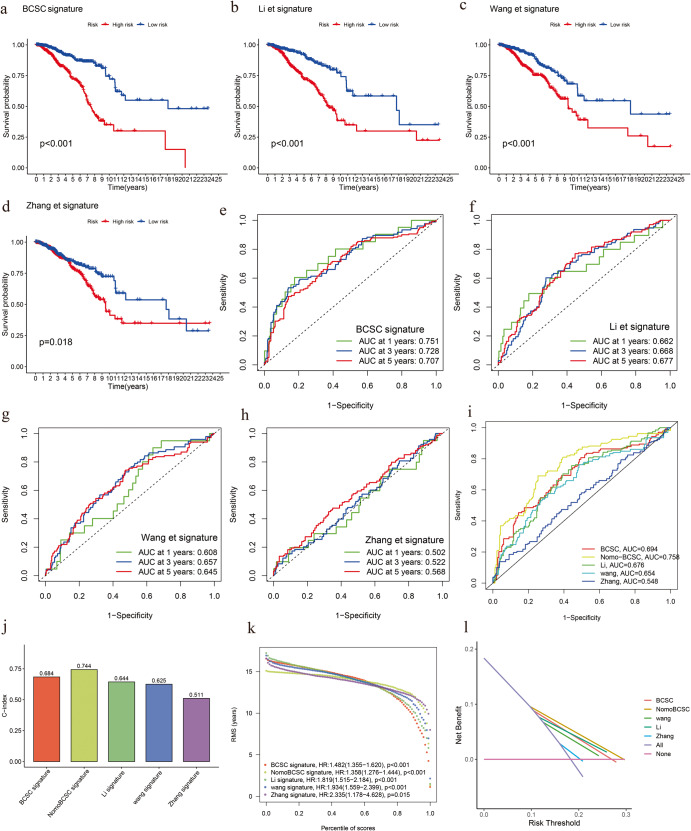
Comparison of the prognostic value of various gene signatures in breast cancer. **a**–**d** Kaplan–Meier survival curves of high- and low-risk patients stratified by BCSCRS, Li et al. signature, Wang et al. signature, and Zhang et al. signature, respectively. **e**–**h** Area under the ROC curve (AUC) of various signatures in predicting 1-, 3-, and 5-year overall survival in breast cancer. **i** Comparison of the AUC of various signatures in predicting overall survival in breast cancer. **j**–**l** C-index (concordance index), RMS, and DCA (Decision Curve Analysis) analysis of various signatures in breast cancer.

While we have demonstrated the accuracy of BCSCRS from various perspectives, the most important aspect of clinical prognostic models is their usefulness in clinical practice. To highlight the advantages of the BCSCRS developed in this study, we compared it with other breast cancer signatures. To minimize data dimensionality and avoid data conflicts in the same direction, we selected three distinct research directions from recently published articles and analyzed and compared their signatures in the entire TCGA cohort. In order to avoid genes involved in the same biological process that might be linked or even screened for duplicate genes, we deliberately chose three models in different directions. The three signatures we selected were associated with breast cancer prognosis; these were a macrophage marker gene signature (Li et al.)^11^, a lactate metabolism-related gene signature (Zhang et al.)^12^, and a ferroptosis-related gene signature (Wang et al.)^13^. The risk score for each breast cancer patient was calculated as per the original method, and all patients in TCGA were divided into high- and low-risk groups according to the median for further survival analysis. The survival curves showed that the low-risk group had better survival (Fig. 6a–d). Except for the Zhang et al. signature (AUC = 0.502, 0.522, 0.568), the other signatures exhibited good potential in predicting breast cancer survival in 1-, 3-, and 5-year intervals based on the area under the receiver operating characteristics curve (Fig. 6e–h). The BCSCRS (AUC = 0.694) and nomograms (AUC = 0.758) developed in this study showed higher accuracy than other signatures (Fig. 6i). The nomograms optimized by clinical variables were not included in the signature comparison but were only used for auxiliary validation. The results of C-index, RMS, and DCA analysis further confirmed the superior accuracy of BCSCRS in predicting the survival of breast cancer (Fig. 6j–l). Collectively, the comparison results highlight the outstanding predictive capabilities of BCSCRS in relation to breast cancer survival, underscoring its potential as a valuable tool in clinical practice. The higher accuracy and robustness of BCSCRS, as supported by multiple evaluation metrics, signify its significant contribution to breast cancer prognosis prediction.

### Analysis of immune landscape in breast cancer based on BCSCRS

**Fig. 7:**
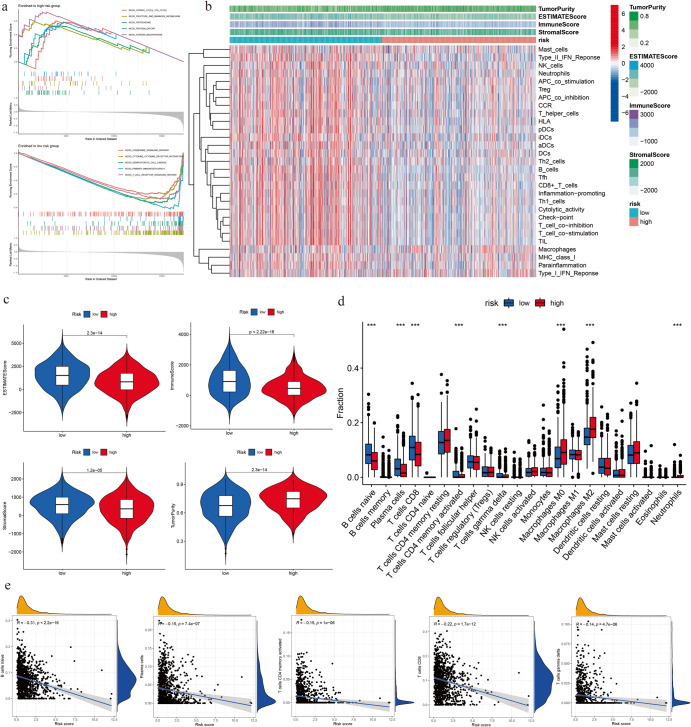
Immune landscape analysis of tumor microenvironment and immune infiltration. **a** GSEA enrichment analysis in high- and low-risk groups. **b** Heat map showing the overall immune landscape in the risk group. **c** Differential analysis of tumor microenvironment between two risk groups. **d** Differential analysis of immune infiltration cells between two risk groups. **e** Correlation analysis between BCSCRS and immune infiltration cells.

Given that a significant correlation between the BCSC core genes and immune activity was observed in our analysis, to further explore this association, we conducted GSVA and GSEA analyses and found marked differences in biological processes between high- and low-risk groups. In the high-risk group, signaling pathways were significantly enriched, including steroid biosynthesis, fructose and mannose metabolism, protein export, proteasome, and citrate cycle TCA cycle. In contrast, the low-risk group was characterized by primary immunodeficiency and T-cell receptor signaling pathway (Fig. 7a), suggesting a stronger connection between the low-risk group and immunity. To further examine this relationship, we investigated the characteristics including TME and immune infiltration related to the immune landscape (Fig. 7b). Results revealed that the ESTIMATE score, immune score, and stromal score of the low-risk group were significantly higher than those of the high-risk group, while tumor purity results were reversed (Fig. 7c). These findings suggest that stromal and immune cell content was higher than that of tumor cells in the TME. Our ssGSEA analysis on immune infiltration showed that the expression levels of cells in the TME in the low-risk group were higher, except for macrophages (Fig. 7b). Using the CIBERSORT algorithm, we analyzed the differences in 22 types of immune cells in the high- and low-risk groups and observed that naive B cells, plasma cells, CD4 memory-activated T cells, CD8 T cells, and gamma delta T cells were more infiltrated in the low-risk group, whereas higher infiltration of immunosuppressive immune cells such as M0 and M2 macrophages was found in the high-risk group (Fig. 7d). Furthermore, the infiltration levels of naive B cells, plasma cells, CD4 memory-activated T cells, CD8 T cells, and gamma delta T cells were negatively correlated with risk score (Fig. 7e). These results suggest a close relationship between BCSCRS and immune cells, with lower risk scores indicating higher expression of stromal cells and immune cells in the TME.

### Evaluation of the immunotherapy response based on BCSCRS

**Fig. 8:**
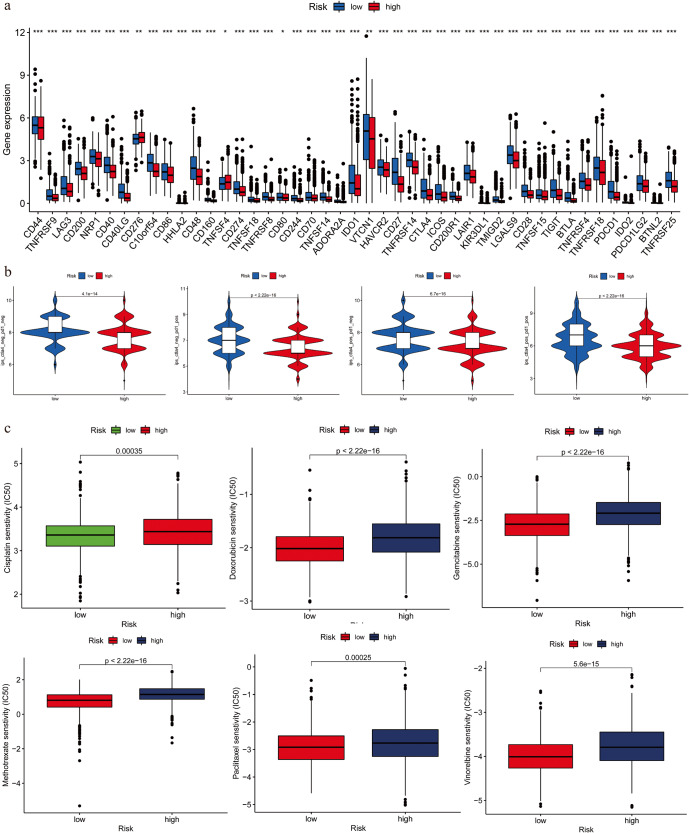
Therapeutic response analysis of immune checkpoint inhibitors. **a** Expression of 27 immune checkpoint molecules. **b** Analysis of IPS (Immunophenotype Score) between two risk groups. **c** The box plot shows the distribution of IC50 values at two risk groups for six common chemotherapy drugs for breast cancer. A lower IC50 value indicates greater drug sensitivity. The upper and lower bounds of the box signify the third and first quartiles, respectively, while the center line within the box represents the median. The whiskers represent the data points, which range within 1.5 times the interquartile distance.

To further examine the association between BCSCRS and immunotherapy response, we assessed several indicators. First, we analyzed the expression of immune checkpoint molecules and found that the low-risk group had significantly higher expression of 27 immune checkpoints, suggesting that these patients might be more responsive to immune checkpoint inhibitors (Fig. 8a). We also used IPS scores of PD1 and CTLA4 as quantitative indicators to further assess the effectiveness of immune checkpoint inhibitors. Our results showed that the IPS-CTLA4, IPS-PD1, and IPS-PD1-CTLA4 scores were significantly higher in the low-risk group, indicating that these patients might have better effectiveness when treated with PD1 and CTLA4 inhibitors (Fig. 8b). We also analyzed the association between BCSCRS and the IC_50_ value of chemotherapy drugs commonly used in breast cancer treatment. Our results showed that the low-risk group was more sensitive to chemotherapy drugs such as Cisplatin, Doxorubicin, Gemcitabine, Methotrexate, Paclitaxel, and Vinorelbine, which suggests that these patients may experience better efficacy and be less likely to develop drug resistance (Fig. 8c). It is worth noting that BCSCs have been shown to be involved in the drug resistance process of breast cancer^14^. Therefore, our findings imply that the low-risk group may have better responses to both immunotherapy and chemotherapy, which could have noteworthy clinical implications.

### The spatial domestication of CD79A^+^CD24^-^PANCK^+^-BCSCs subpopulation cells and exhausted CD8^+^T cells in the tumor microenvironment

**Fig. 9:**
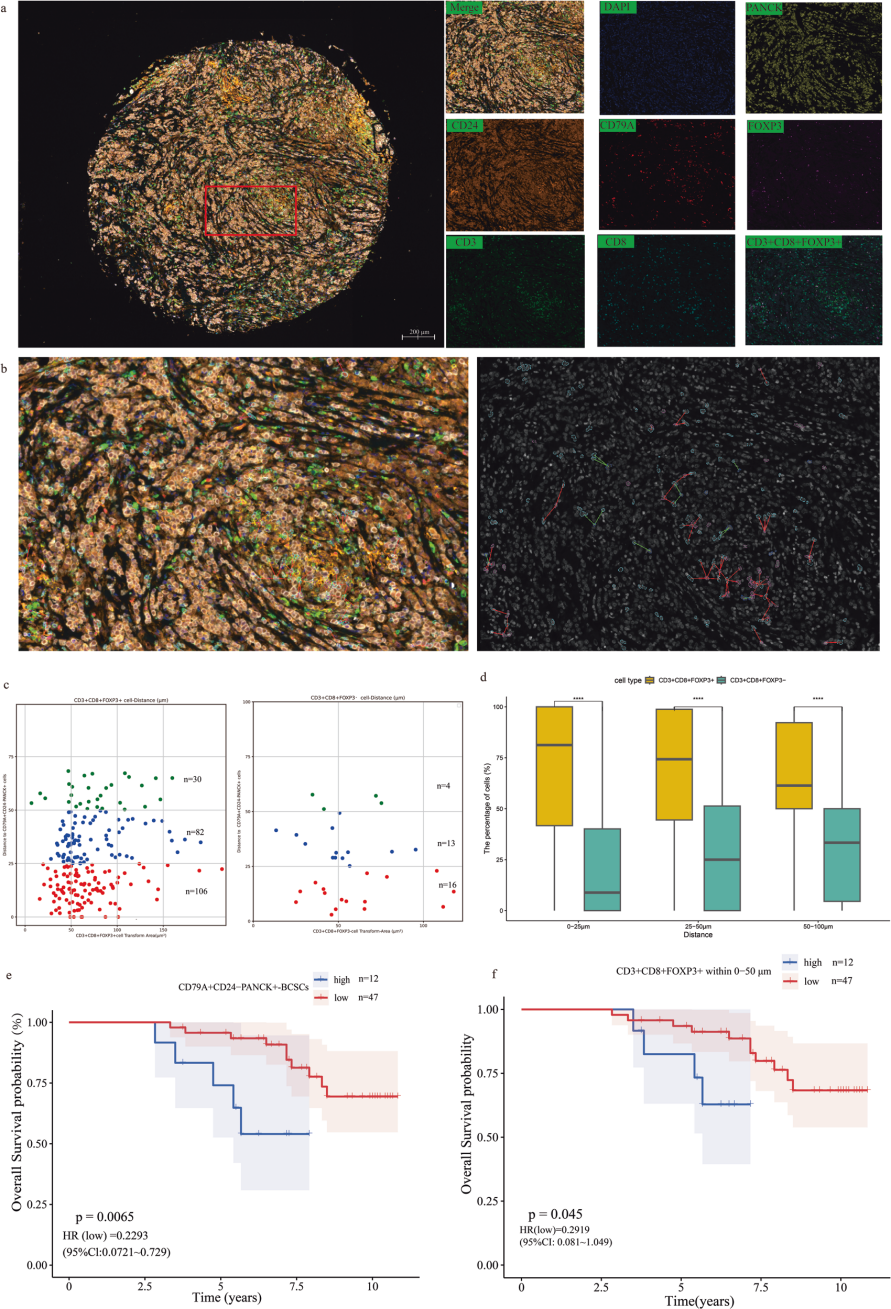
TissueFAXS Cytometry panoramic tissue quantitative analysis described the spatial distribution of CD79A^+^CD24^-^PANCK^+^-BCSCs subpopulation and different subtypes of CD8^+^T cells in the TME. **a** Representative multi-label staining in samples from breast cancer patients: DAPI (Bluish violet), CD3 (green), CD8 (blue), FOXP3 (purple), PANCK (yellow), CD24 (orange), CD79A (red). **b** Schematic diagram of spatial proximity analysis of representative areas (Left: Original image of spatial proximity analysis. Right: Simulation picture of spatial proximity analysis). **c** Representation of the spatial distribution of CD3^+^CD8^+^FOXP3^+^ and CD3^+^CD8^+^FOXP3^-^ T cells within the distance gradients of CD79A^+^CD24^-^PANCK^+^-BCSCs subpopulation (0–25 μm, 25–50 μm, 50–100 μm). **d** Box plot of differences between CD3^+^CD8^+^FOXP3^+^T cells and CD3^+^CD8^+^FOXP3^-^T cells within the distance gradients of CD79A^+^CD24^-^PANCK^+^ cell subsets (0–25 μm, 25–50 μm, 50–100 μm) in total TMA (The horizontal coordinate represents the distance gradient, the ordinate indicates the proportion of such cells). The upper and lower bounds of the box signify the third and first quartiles, respectively, while the center line within the box represents the median. The whiskers represent the data points, which range within 1.5 times the interquartile distance. **e** Survival curve based on the number of CD79A^+^CD24^-^PANCK^+^-BCSCs subpopulation (cutoff = 34.5). **f** Survival curve based on the number of CD3^+^CD8^+^FOXP3^+^ T cells within 50 μm (cutoff = 103). *p < 0.05; **p < 0.01; ***p < 0.001.

Among the ten genes used to construct a risk score model, Pearson correlation analysis revealed that CD24 and CD79A is the most positively and negatively correlated with BCSCRS (Supplementary Fig. 4a). The results of Protein-protein interaction (PPI) analysis showed that CD79A and CD24 has a potential interaction (Supplementary Fig. 4b). Previous studies revealed that CD79A^+^ plays an important role in maintaining cells pluripotency and promoting malignant cells infiltration with poor clinical prognostic^15,16^. Additionally, CD79A gene was searched out in the Genecards database as keywords “breast cancer stem cells” and our subsequent data analysis also revealed that it is indeed an important gene involved in breast cancer cell stemness. Additionally, CD24^-^ is a well-known BCSCs marker^17^. Based on above evidences, we had a strong desire to investigate the effects of the BCSC population with CD79A^+^ and CD24^-^ on tumor immune microenvironment. All we know that CD8^+^T cells are an important component of tumor immune microenvironment, and its exhausted or not play pivotal roles in tumor immunotherapy response^18,19^. Studies revealed that CD8^+^T-cell exhausting is a dynamic process and only completely exhausted CD8^+^T cells totally lose its function of killing tumor cells^20,21^. The most recent studies showed that FOXP3^+^ is a marker of CD8 + T-cell completely exhausted and it is entirely induced by the tumor immune microenvironment which can strongly indicate that the immunosuppressive domestication of CD8^+^T cells by a certain tumor cell subpopulation^22^. Therefore, we would like to explore the spatial relationship between CD79A^+^CD24^-^PANCK^+^-BCSCs subpopulation and CD8^+^ T cells with FOXP3^+^ or not to reveal the influence of CD79A^+^CD24^-^PANCK^+^-BCSCs subpopulation on CD8^+^T cells and tumor microenvironment. After eliminating the poorly stained samples, we used multiplex immunofluorescence staining and TissueFAXS Cytometry Panoramic Tissue Quantification assays for follow-up analysis (Fig. 9a, b). Finally, we found that CD79A^+^CD24^-^PANCK^+^-BCSCs subpopulation was present in 59 of the samples (Table 3) detected with CD24^-^PANCK^+^ (Supplementary Fig. 4c), which accounted for 1.09% of all breast cancer cells and setting them as the center, within 100 μm (Define strong interactions between cells), completely exhausted CD8^+^FOXP3^+^ T cells accounted for the majority of the total CD8^+^ T cells (Fig. 9c), the proportions are respectively 65.7% (0–25 μm), 67.2% (25–50 μm) and 65.6% (50–100 μm), strongly suggesting the immunosuppressive domestication effect of CD79A^+^CD24^-^PANCK^+^ on CD8^+^ T cells (Fig. 9d). We also evaluated the effects of CD79A^+^CD24^-^PANCK^+^-BCSCs subpopulation and CD8^+^FOXP3^+^ T cells on breast cancer patients survive status, finding that both CD79A^+^CD24^-^PANCK^+^-BCSCs cells-High and CD8^+^FOXP3^+^ cells-High (within 50 μm to CD79A^+^CD24^-^PANCK^+^-BCSCs subpopulation) had poorer survival probability (Fig. 9e, f), further indicating that these two groups of cells contribute to poor prognosis may due to the tumor immunosuppressive microenvironment they shaped.

**Table 3:** Detailed clinical information of the spatial proximity analysis cohort.

Variables	59 samples in TMA
Age	
≥65 years	16
<65 years	43
Survival state	
alive	44
dead	15
M classification	
M0	59
M1	NA
N classification	
N0	28
N1	10
N2	17
N3	4
T classification	
T1	11
T2	43
T3	5

## Discussion

Breast cancer is a highly heterogeneous malignancy occurring in breast tissue^23^. Although surgery, chemotherapy, radiotherapy, and emerging immunotherapy approaches have significantly improved prognosis, the heterogeneity of breast cancer resulting in breast cancer recurrence, metastasis, drug resistance, and immune escape still significantly reduces the survival rate of breast cancer patients^24,25^. Thus, fully understanding the heterogeneity of breast cancer and using its characteristics in clinical diagnosis and treatment will help further improve the clinical benefits of breast cancer patients. Recent studies have revealed that breast cancer stem cells are the origin of heterogeneity^26,27^. However, few studies have been performed to explore the potential impact of tumor stem-cell-related characteristics on breast cancer typing and immune landscape^2^. In this study, we demonstrate that cancer stem-cell-related genes can be used for classifying breast cancer and develop and identify a breast cancer stem-cells-related risk panel that sheds light on the immune landscape of breast cancer for personalized immunotherapy. In addition, the accuracy and robustness of BCSCRS constructed in this study were superior to the other three breast cancer prognosis models used for comparison, which has important reference value for its clinical application.

In present research, breast cancer patients can be divided into two subtypes. The results show that Cluster 1 is significantly enriched in the signaling pathways associated with immune activity, such as the T-cell receptor signaling pathway and the B-cell receptor signaling pathway, suggesting that Cluster 1 may have higher immune activity. The strength of the T-cell receptor (TCR) signal is a key determinant of T-cell response, and the affinity of the interaction between the T-cell receptor and the peptide-bound MHC directly determines the frequency and rate of activation of naive T cells^28,29^. Additionally, the immune infiltration of B lineage, CD8 T-cell, Cytotoxic lymphocytes, Myeloid dendritic cells, NK cells, and T cells are more abundant in Cluster 1. Interestingly, the above immune cells with anti-tumor effects also had a greater abundance of infiltration in the low-risk group. It has been reported that B cells not only play an important role in CRT-T immunotherapy, but also serve as antigen-presenting cells to initiate CD4^+^ and CD8^+^ T cells^30^. The activity of tumor-infiltrating CD8^+^ T cells and natural killer (NK) cells, which are important effector cells against tumor cells, is significantly inhibited by immunosuppressive cytokines and tumor-associated macrophages (TAMs) in the tumor microenvironment^31,32^. Currently, some dendritic cell-based vaccines can effectively improve the survival rate of patients by specifically increasing the secretion of cytokines in CD8^+^ effector T cells and NK cells^33,34^. Dendritic cells have been demonstrated to be the most important professional antigen-presenting cells (APCs), which can specifically stimulate the maturation of B cells and T cells to initiate an acquired immune response^35^. The primary function of myeloid dendritic cells is to process the captured antigen and then present it to the antigen surface via a major histocompatibility complex^36^. In addition to the immune cells with anti-tumor activity mentioned above, there is also a class of immunosuppressive cells in the tumor microenvironment that deserve attention. Specifically, M0 and M2 macrophages were found to have significantly high expression of infiltration abundance in the high-risk group and were significantly positively correlated with BCSCRS in this study. As a very important immune cell in normal human body, macrophages are believed to transmit immune signals, phagocytose antigens, and clear abnormal cells in the body. Recent studies have confirmed that M2 macrophages, which exist in large numbers in the tumor microenvironment, can evade T-cell-mediated immune surveillance by inducing the upregulation of PD-L1 and promote the progression of breast cancer by promoting angiogenesis, immune escape, and immunosuppression^37,38^, while M0 macrophages are closely associated with distal metastasis of tumor cells and poor prognosis^39^. These results suggest that immunoactivity is higher in the low-risk group, and therefore a better response may be achieved when receiving immunotherapy. The synergy between the risk score model and the complex immune landscape provides clinicians with a comprehensive framework for improved decision-making and improved prognostic accuracy, and this study has important clinical implications for the development of personalized immunotherapy strategies for breast cancer patients.

Furthermore, a breast cancer stem cell subpopulation strongly associated with poor prognosis has been identified, which has been defined as CD79A^+^CD24^-^PANCK^+^-BCSCs subpopulation. Moreover, a stronger interaction was found between the tumor stem cell subpopulation and exhausted CD8^+^ T cells with FOXP3^+^ using multiple immunofluorescence techniques. CD8^+^FOXP3^+^ T cells are a class of exhausted CD8^+^ T cells with Treg-like and cytolytic properties^40^, in the process of exhaustion of tumor-infiltrating CD8^+^T cells induced by the tumor microenvironment^22^. Although the current study shows that CD8^+^FOXP3^+^ T cells are a special class of exhausted CD8^+^ T cells, the specific mechanism of action of tumor microenvironment inducing CD8^+^ T-cell exhaustion is not clear. The TME is a complex dynamic ecosystem composed of various cell types, extracellular matrix (ECM), blood vessels, and signaling molecules that play a critical role in tumor initiation, progression, and therapeutic response^41^. Although the specific regulatory mechanism between cancer stem cells and immune cells has not been fully clarified, current studies have confirmed that cancer stem cells promote the recruitment of immunosuppressive cells such as Tregs to TME by producing immunosuppressive factors in the tumor microenvironment^42^. Tregs play a critical role in maintaining immune tolerance and preventing autoimmune reactions, and in TME, their presence can shape the tumor suppressor microenvironment by inhibiting the activity of effector T cells (such as cytotoxic T cells) and other immune cells. However, it is clear that the CD79A^+^CD24^-^PANCK^+^-BCSCs subpopulation identified in this study does not recruit exhausted CD8^+^ T cells with FOXP3^+^ in this manner, as CD8^+^FOXP3^+^T cells are a specific class of exhausted CD8^+^ T cells that are difficult to detect in both blood and normal tissues^22^. This suggests that CD79A^+^CD24^-^PANCK^+^-BCSCs subpopulation may secrete some chemokines or cytokines to induce the exhaustion of CD8^+^ T cells to overexpress FOXP3. This similar domestication relationship between breast cancer stem cells and exhausted CD8^+^ T cells allows us to understand the role of breast cancer stem cells in shaping the immunosuppressive microenvironment. However, the specific mechanism remains to be further explored.

Despite the noteworthy findings and contributions of our study, there are several limitations that need to be acknowledged. First, the data used in our study came from multiple databases with varying sequencing methods and depths, which may have affected the level of gene detection and introduced bias. Second, the heterogeneity of tumors among various patients is an inherent limitation of our study. Although we found evidence of a relationship between breast cancer stem cells and exhausted CD8^+^ T cells, further biological experiments are needed to elucidate the specific mechanisms involved. Finally, the selection of datasets was limited by the availability of clinical data, which may have resulted in selection bias. Further studies with larger and more diverse datasets, and more rigorous experimental designs, are needed to validate our findings and advance the understanding of the relationship between breast cancer stem cells and the immune microenvironment.

In conclusion, our study indicates that BCSCs-related subtypes and BCSCRS could be useful biomarkers for exploring the heterogeneity of breast tumors and predicting their immunotherapy reactivity. Notably, the CD79A^+^CD24^-^PANCK^+^-BCSCs subpopulation with poor breast cancer prognosis in this study was strongly associated with CD8^+^ T-cell exhaustion and the formation of an immunosuppressive tumor microenvironment. In the process of immunotherapy, the tumor microenvironment can be remodeled by targeting elimination of CD79A^+^CD24^-^PANCK^+^-BCSCs subpopulation or reversing the exhaustion of CD8^+^ T-cell, so as to restore the anti-tumor effect of effector T-cell. However, further investigations are necessary to fully understand the underlying mechanisms.

## Methods

### Public datasets for breast cancer stem cell analysis

Gene expression data, somatic mutation data, gene mapping file, and clinical phenotypic data of breast cancer were sourced from the GDC-TCGA-BRCA project in the UCSC (University of California Santa Cruz) Genome Browser database (https://xenabrowser.net/datapages/) and Gene Expression Omnibus (GEO) database (https://www.ncbi.nlm.nih.gov/geo/)^43–45^. After excluding normal tissue samples and samples from the same patient, complete information for 1069 patients in The Cancer Genome Atlas (TCGA) database was obtained. Subsequently, the TCGA patients were randomly divided into a training cohort (*N* = 749) and a test cohort (*N* = 320) to construct the model using the createDataPartition function in the caret package with a 7:3 ratio, which was additionally validated with an external cohort of 327 patients from GSE20685 to verify accuracy and robustness of the model. Detailed description of all cohorts can be found in Table 4. Finally, breast cancer stem-cells-related genes (BCSCGs) were collected from the GeneCards database (https://www.genecards.org/) and the results filtered by setting a relevance score higher than 30^46^.

**Table 4:** The clinical characteristics of breast cancer in TCGA cohort and GSE20685.

Variables	TCGA train cohort (*N* = 749)	TCGA test cohort (*N* = 320)	GSE cohort (*N* = 327)
Incomplete	*N* = 126	*N* = 51	0
Age			
≥65 years	169	78	22
<65 years	454	191	305
Sex			
Female	614	268	327
Male	9	1	0
M classification			
M0	615	261	244
M1	8	8	83
N classification			
N0	306	132	122
N1	211	87	102
N2	70	33	63
N3	36	17	40
T classification			
T1	160	75	101
T2	373	155	188
T3	68	30	30
T4	22	9	8
Stage classification			
Stage I	113	46	NA
Stage II	363	158	NA
Stage III	139	57	NA
Stage IV	8	8	NA

### Identifying breast cancer stem cell subtypes through unsupervised clustering analysis of BCSCs

The non-negative matrix factorization (NMF) algorithm was used to identify BCSC-related subtypes and their prognosis^47,48^. Initially, the expression data underwent dimensionality reduction through univariate Cox analysis. Subsequently, patients were categorized into distinct clusters based on the gene expression using the NMF package. The distribution of various breast cancer stem-cells-related subtypes was visualized using principal component analysis (PCA), and a Sankey diagram was utilized to illustrate the relationship between different clusters, tumor mutation burden (TMB), and survival status. Survival analysis was conducted using Kaplan–Meier method and the findings were visually represented using the Survminer R package. The tumor microenvironment (TME) and immune infiltration in various clusters were quantified by ESTIMATE and MCPcounter packages^49,50^, respectively. The GSVA package was used to obtain the gene sets of “c2.cp.kegg.v7.4.symbols.gmt” from the MSigDB database (https://www.gsea-msigdb.org/gsea/msigdb/index.jsp) for the normalized enrichment score (NES) of KEGG (Kyoto Encyclopedia of Genes and Genomes) pathways among TCGA patients^51,52^.

### Differential expression analysis of breast cancer stem-cell-related subtypes

To identify differential and core genes of distinct BCSCs-related clusters, we conducted differential expression analysis and WGCNA after unsupervised clustering of all TCGA breast cancer samples. Differential genes among BCSCs-related subtypes were analyzed using the limma package with a logFC filter of 1 and a *p*-value cutoff of 0.05^53^ and visualized using pheatmap and ggplot2 packages. The WGCNA package was used to explore hub genes for weighted gene co-expression network analysis^54^. Differential and core genes were subjected to Gene Ontology (GO) and Kyoto Encyclopedia of Genes and Genomes (KEGG) enrichment analysis using the clusterProfiler R package^55,56^.

### Development and validation of a BCSC-related prognostic signature

Least absolute shrinkage selection operator (LASSO) Cox regression was used to select BCSCGs for predicting the survival and prognosis of breast cancer^57^, and BCSCGs associated with prognosis with minimized lambda were selected. A prognostic model based on these BCSCGs was constructed through multivariate Cox regression analysis in the TCGA training cohort (*N* = 749). To validate the prognostic model, the TCGA test cohort (*N* = 320) and GSE20685 cohort (*N* = 327) were used as internal and external validation cohorts, respectively. The accuracy of the prognosis model was evaluated by calculating the risk formula, as shown below:

$${\rm{Risk}}\,{\rm{score}}\,\left({\rm{BCSCRS}}\right)=\mathop{\sum }\limits_{i=1}^{n}({Expressioni}\times {coefi})$$

Then, the risk score for each sample was calculated by the predict function in the survival package, based on the expression of genes and their corresponding regression coefficients in the model formula. Patients were divided into high-risk and low-risk groups according to the median risk score. The accuracy of the Cox regression model was assessed by generating receiver operating characteristic (ROC) curves and calculating the area under the curve (AUC) values, using the timeROC package^58^. The pheatmap package was used to plot the risk curves for all cohorts and survival status maps for all patients. The overall survival (OS) status between high-risk and low-risk groups was compared using Kaplan–Meier analysis, which was performed using the survminer R package.

### Correlation analysis between BCSCRS and clinical variables

Correlation analysis was performed between the BCSCRS and clinical variables such as age, gender, stage, and TNM stage. Age was dichotomized into two groups based on the standard of 65 years old, while M stage was classified as M0 and M1. Gender was categorized as male or female. However, stage, N, and T were divided into four groups as per the requirements. The differences in clinical variables were analyzed using the limma package, and the results were visualized using the ggpubr package.

### Building a nomogram for prognostic risk assessment

To improve the precision of the prognostic model, a nomogram was developed that incorporated the risk score and clinical variables such as age and tumor stage. Initially, univariate, and multivariate Cox regression analyses were conducted to assess whether the risk scores and clinical variables could serve as independent prognostic factors. Next, the rms package was used to construct the nomogram and calibration curve, which included patient age, gender, TNM stage, and risk score^59^. To compare the predictive accuracy of the nomogram with other prognostic factors, Receiver Operating Characteristic (ROC) and Decision Curve Analysis (DCA) were performed using the timeROC and ggDCA package, respectively.

### Evaluating the prognostic accuracy of BCSCRS against established models

To validate the proposed prognostic model for breast cancer, a comparative analysis was conducted against three distinct prognostic models. The first model was a ferroptosis-related signature developed by Wang et al.^13^, the second was a macrophage marker genes signature in breast cancer constructed by Li et al.^11^, and the third was a lactate metabolism-related prognostic model proposed by Zhang et al.^12^. To maintain consistency with the literature and reduce data dimensionality, gene expression levels were extracted for each model and multivariate Cox regression was performed to obtain the regression coefficients of each gene. Subsequently, risk scores were calculated for each sample, and the predictive power and clinical utility of each model were assessed using the concordance index (C-index) and DCA, as well as the ROC curves and survival analysis. All analyses were performed using the timeROC and survival packages in R software.

### Estimating BCSCRS on tumor microenvironment

Gene set enrichment analysis (GSEA) was conducted to explore the biological functions of different risk groups^60^. Considering the pivotal role of the TME in tumor immunotherapy^61,62^, the ESTIMATE package was used to analyze the composition of the tumor microenvironment^49^. The CIBERSORT algorithm was utilized to analyze transcriptome data and obtain the expression levels of 22 types of immune cells in each sample^63,64^. In addition to the differences in immune checkpoint expression, immune score, and immune cell infiltration among various risk groups, the correlation between immune cells and risk score was also analyzed.

### Assessment of immunotherapy and chemotherapy response

In addition to analyzing the immune characteristic, we also investigated the responsiveness of various risk groups to immune checkpoint inhibitor therapy and commonly used chemotherapy drugs for breast cancer. The Immunophenotype Score (IPS), a good predictor of CTLA4 (Cytotoxic T Lymphocyte-Associated Antigen-4) and PD1 (Programmed Death 1) responsiveness, was obtained from the TCIA database (https://tcia.at/) and utilized to predict the responsiveness of high- and low-risk groups during immune checkpoint inhibitor therapy^65^. To predict chemosensitivity, the 50% maximal inhibitory concentration (IC50) for each sample was calculated using the R package “pRRophetic”^66^, which offers a comprehensive set of pre-trained predictive models that harness gene expression data to make accurate projections of drug responses. Specifically, the drug code corresponding to the target compound within the database was selected, and the gene expression matrix of breast cancer from the TCGA dataset was employed as the designated input file. The prediction of IC50 values for the identified drug was achieved through the utilization of the predictProfileIC50 function.

### Patients and tissue samples

In this study, a total of 267 patients with breast cancer were included. The tissue microarray comprising 267 tumor samples from these patients, along with patient clinical data, was directly retrieved from Shanghai Outdo Biotech Company in accordance with relevant regulations. The study was conducted in compliance with the Declaration of Helsinki. We confirm that written informed consent was obtained from all patients involved in the study, ensuring their voluntary participation, and the use of human tumor tissue was approved by the Ethics Committee of Shanghai Outdo Biotech Company (approval No.YBM-05-01 and YBM-05-02).

### Immunofluorescence staining and image acquisition

A total of 267 samples of TMAs without drug treatment were selected for TissueFAXS panoramic tissue quantitative assay, which Multiplex immunofluorescence staining of tissues was conducted using the Alpha TSA 7-color fluorescence staining kit (Alpha TSA Multiplex IHC Kit) sourced from Beijing, China. Specifically, XTSA 480 (Cat: ZA0508), XTSA 520 (Cat: ZA0293), XTSA 570 (Cat: HA720082), XTSA 620 (0804-3), XTSA 690 (ab20034), and XTSA 780 (ZM0069) were employed for the labeling of CD8, CD79A, CD3, CD24, FOXP3, and PANCK, respectively. Briefly, tissue microarray (TMA) removes residual paraffin with xylene and anhydrous ethanol and rehydrates it with ethanol of different concentration gradients. Following this, the sample underwent two rounds of 5-minute rinses with distilled water, followed by microwave repair using the antigen repair solution provided in the kit. After cooling, the sample was rinsed thrice with PBST and immersed in sealing liquid at room temperature for 15 min. Subsequent steps included the incubation of diluted primary antibodies at 37 °C for 1 h, followed by a wash with PBST three times. TMA and corresponding secondary antibodies were then incubated at 37 °C for 10 min, washed thrice with PBST, and treated with fluorescent dye for 5 min at room temperature. This dyeing process was repeated to ensure complete labeling of all relevant markers. Finally, nuclear dye (DAPI) was applied for 8 min at room temperature. After rinsing with PBST, the slide was sealed for subsequent image scanning. The ZEISS Axioscan7 full-slice imaging system was employed for image acquisition, with ZEN 3.3 software used for image analysis. For quantitative analysis, Strata Quest software (TissueGnostics) was used to calculate parameters such as nuclear area, fluorescence intensity, and cell density per cell area for identifying positive cells. This software was also used to quantitatively count CD3^+^CD8^+^FOXP3^+^ T and CD3^+^CD8^+^FOXP3^-^ T cells based on a distance gradient ranging from 0–25 μm, 25–50 μm, and 50–100 μm from CD79A^+^CD24^-^PANCK^+^ cells^67^.

### Statistical analysis

Statistical analyses in this study were performed using R software (version 4.0.3 and 4.1.3) and relevant R packages sourced from Bioconductor and CRAN. The Wilcoxon test was used to compare differences between two groups, while Kruskal–Wallis test was utilized for comparisons involving more than two groups. Correlation analyses were conducted using Pearson test. Median values were used for all truncation values relating to grouping. Statistical significance was determined as a *P-*value < 0.05.

### Reporting summary

Further information on research design is available in the Nature Research Reporting Summary linked to this article.

### Supplementary information

Supplementary Information

Reporting Summary

## Data Availability

All raw data used in this work can be acquired from GDC-TCGA-BRCA project in the UCSC Genome Browser database (https://xenabrowser.net/datapages/) and the Gene Expression Omnibus (GEO) (https://www.ncbi.nlm.nih.gov/geo/) with accession number GSE20685. Additional data and materials are available from the University of California, Santa Cruz (UCSC) Xenabrowser (https://xenabrowser.net/). These data are currently publicly available.

## Code availability

Data analysis was performed by R software (version 4.0.3 and 4.1.3) and specific code, and the corresponding parameter settings were set in the code. The code used for analysis can be accessed by the corresponding author on reasonable request, and the corresponding code for this article is stored in the below link (https://www.jianguoyun.com/p/DX9xHisQ5rKeCxivxPwEIAA).

## References

[1] Sung H (2021). Global cancer statistics 2020: GLOBOCAN estimates of incidence and mortality worldwide for 36 cancers in 185 countries. CA Cancer J. Clin..

[2] Cummings MC, Chambers R, Simpson PT, Lakhani SR (2011). Molecular classification of breast cancer: is it time to pack up our microscopes?. Pathology.

[3] Polyak K (2011). Heterogeneity in breast cancer. J. Clin. Invest..

[4] Zeng X (2021). Breast cancer stem cells, heterogeneity, targeting therapies and therapeutic implications. Pharm. Res..

[5] Clarke MF (2019). Clinical and therapeutic implications of cancer stem cells. N. Engl. J. Med..

[6] Zhang Z, Chen X, Zhang J, Dai X (2021). Cancer stem cell transcriptome landscape reveals biomarkers driving breast carcinoma heterogeneity. Breast Cancer Res. Treat..

[7] Nedeljkovic M, Damjanovic A (2019). Mechanisms of chemotherapy resistance in triple-negative breast cancer-how we can rise to the challenge. Cells.

[8] Riggio AI, Varley KE, Welm AL (2021). The lingering mysteries of metastatic recurrence in breast cancer. Br. J. Cancer.

[9] Quaglino E, Conti L, Cavallo F (2020). Breast cancer stem cell antigens as targets for immunotherapy. Semin. Immunol..

[10] Samstein RM (2019). Tumor mutational load predicts survival after immunotherapy across multiple cancer types. Nat. Genet..

[11] Li Y, Zhao X, Liu Q, Liu Y (2021). Bioinformatics reveal macrophages marker genes signature in breast cancer to predict prognosis. Ann. Med..

[12] Zhang Z, Fang T, Lv Y (2022). A novel lactate metabolism-related signature predicts prognosis and tumor immune microenvironment of breast cancer. Front. Genet..

[13] Wang D (2021). Identification of the prognostic value of ferroptosis-related gene signature in breast cancer patients. BMC Cancer.

[14] Palomeras S, Ruiz-Martinez S, Puig T (2018). Targeting breast cancer stem cells to overcome treatment resistance. Molecules.

[15] Lenk L (2021). CD79a promotes CNS-infiltration and leukemia engraftment in pediatric B-cell precursor acute lymphoblastic leukemia. Commun. Biol..

[16] Reynaud D, Lefort N, Manie E, Coulombel L, Levy Y (2003). In vitro identification of human pro-B cells that give rise to macrophages, natural killer cells, and T cells. Blood.

[17] El Abbass KA (2020). The role of breast cancer stem cells and some related molecular biomarkers in metastatic and nonmetastatic breast cancer. Clin. Breast Cancer.

[18] Park J, Hsueh PC, Li Z, Ho PC (2023). Microenvironment-driven metabolic adaptations guiding CD8(+) T cell anti-tumor immunity. Immunity.

[19] Farhood B, Najafi M, Mortezaee K (2019). CD8(+) cytotoxic T lymphocytes in cancer immunotherapy: a review. J. Cell Physiol..

[20] Rancan C (2023). Exhausted intratumoral Vdelta2(-) gammadelta T cells in human kidney cancer retain effector function. Nat. Immunol..

[21] Steele MM (2023). T cell egress via lymphatic vessels is tuned by antigen encounter and limits tumor control. Nat. Immunol..

[22] Zheng C (2017). Landscape of infiltrating T cells in liver cancer revealed by single-cell sequencing. Cell.

[23] Roulot A (2016). Tumoral heterogeneity of breast cancer. Ann. Biol. Clin..

[24] Prat A (2015). Clinical implications of the intrinsic molecular subtypes of breast cancer. Breast.

[25] Tsang JYS, Tse GM (2020). Molecular classification of breast cancer. Adv. Anat. Pathol..

[26] Bai X, Ni J, Beretov J, Graham P, Li Y (2018). Cancer stem cell in breast cancer therapeutic resistance. Cancer Treat. Rev..

[27] Nahas GR, Patel SA, Bliss SA, Rameshwar P (2012). Can breast cancer stem cells evade the immune system?. Curr. Med. Chem..

[28] Skokos D (2007). Peptide-MHC potency governs dynamic interactions between T cells and dendritic cells in lymph nodes. Nat. Immunol..

[29] Zehn D, Lee SY, Bevan MJ (2009). Complete but curtailed T-cell response to very low-affinity antigen. Nature.

[30] Wei G, Wang J, Huang H, Zhao Y (2017). Novel immunotherapies for adult patients with B-lineage acute lymphoblastic leukemia. J. Hematol. Oncol..

[31] Doedens AL (2010). Macrophage expression of hypoxia-inducible factor-1 alpha suppresses T-cell function and promotes tumor progression. Cancer Res..

[32] Kawaguchi K (2019). Alteration of specific cytokine expression patterns in patients with breast cancer. Sci. Rep..

[33] Czerniecki BJ (2007). Targeting HER-2/neu in early breast cancer development using dendritic cells with staged interleukin-12 burst secretion. Cancer Res..

[34] Svane IM (2004). Vaccination with p53-peptide-pulsed dendritic cells, of patients with advanced breast cancer: report from a phase I study. Cancer Immunol. Immunother..

[35] Gabrilovich D (2004). Mechanisms and functional significance of tumour-induced dendritic-cell defects. Nat. Rev. Immunol..

[36] Strioga M (2013). Dendritic cells and their role in tumor immunosurveillance. Innate Immun..

[37] Fang W (2021). Progranulin induces immune escape in breast cancer via up-regulating PD-L1 expression on tumor-associated macrophages (TAMs) and promoting CD8(+) T cell exclusion. J. Exp. Clin. Cancer Res..

[38] Santoni M (2018). Triple negative breast cancer: key role of Tumor-Associated Macrophages in regulating the activity of anti-PD-1/PD-L1 agents. Biochim. Biophys. Acta Rev. Cancer.

[39] Huang L (2020). EFEMP2 indicates assembly of M0 macrophage and more malignant phenotypes of glioma. Aging.

[40] Frisullo G (2010). CD8(+)Foxp3(+) T cells in peripheral blood of relapsing-remitting multiple sclerosis patients. Hum. Immunol..

[41] Kise K, Kinugasa-Katayama Y, Takakura N (2016). Tumor microenvironment for cancer stem cells. Adv. Drug Deliv. Rev..

[42] Vahidian F (2019). Interactions between cancer stem cells, immune system and some environmental components: Friends or foes?. Immunol. Lett..

[43] Navarro Gonzalez J (2021). The UCSC Genome Browser database: 2021 update. Nucleic Acids Res..

[44] Wang Z, Jensen MA, Zenklusen JC (2016). A practical guide to The Cancer Genome Atlas (TCGA). Methods Mol. Biol..

[45] Barrett T (2013). NCBI GEO: archive for functional genomics data sets–update. Nucleic Acids Res..

[46] Stelzer G (2016). The genecards suite: from gene data mining to disease genome sequence analyses. Curr. Protoc. Bioinforma..

[47] Gong J (2021). HCC subtypes based on the activity changes of immunologic and hallmark gene sets in tumor and nontumor tissues. Brief. Bioinform..

[48] Sun Z (2022). Identification and validation of an anoikis-associated gene signature to predict clinical character, stemness, IDH mutation, and immune filtration in glioblastoma. Front. Immunol..

[49] Yoshihara K (2013). Inferring tumour purity and stromal and immune cell admixture from expression data. Nat. Commun..

[50] Becht E (2016). Estimating the population abundance of tissue-infiltrating immune and stromal cell populations using gene expression. Genome Biol..

[51] Hanzelmann S, Castelo R, Guinney J (2013). GSVA: gene set variation analysis for microarray and RNA-seq data. BMC Bioinforma..

[52] Liberzon A (2015). The Molecular Signatures Database (MSigDB) hallmark gene set collection. Cell Syst..

[53] Ritchie ME (2015). limma powers differential expression analyses for RNA-sequencing and microarray studies. Nucleic Acids Res..

[54] Langfelder P, Horvath S (2008). WGCNA: an R package for weighted correlation network analysis. BMC Bioinforma..

[55] Yu G, Wang LG, Han Y, He QY (2012). clusterProfiler: an R package for comparing biological themes among gene clusters. OMICS.

[56] Kanehisa M, Goto S (2000). KEGG: kyoto encyclopedia of genes and genomes. Nucleic Acids Res..

[57] Simon N, Friedman J, Hastie T, Tibshirani R (2011). Regularization paths for Cox’s proportional hazards model via coordinate descent. J. Stat. Softw..

[58] Obuchowski NA, Bullen JA (2018). Receiver operating characteristic (ROC) curves: review of methods with applications in diagnostic medicine. Phys. Med. Biol..

[59] Jin C (2017). A nomogram for predicting the risk of invasive pulmonary adenocarcinoma for patients with solitary peripheral subsolid nodules. J. Thorac. Cardiovasc. Surg..

[60] Subramanian A (2005). Gene set enrichment analysis: a knowledge-based approach for interpreting genome-wide expression profiles. Proc. Natl Acad. Sci. USA.

[61] Anderson NM, Simon MC (2020). The tumor microenvironment. Curr. Biol..

[62] Frankel T, Lanfranca MP, Zou W (2017). The role of tumor microenvironment in cancer immunotherapy. Adv. Exp. Med. Biol..

[63] Chen B, Khodadoust MS, Liu CL, Newman AM, Alizadeh AA (2018). Profiling tumor infiltrating immune cells with CIBERSORT. Methods Mol. Biol..

[64] Newman AM (2015). Robust enumeration of cell subsets from tissue expression profiles. Nat. Methods.

[65] Charoentong P (2017). Pan-cancer immunogenomic analyses reveal genotype-immunophenotype relationships and predictors of response to checkpoint blockade. Cell Rep..

[66] Geeleher P, Cox N, Huang RS (2014). pRRophetic: an R package for prediction of clinical chemotherapeutic response from tumor gene expression levels. PLoS ONE.

[67] Li H (2023). S100A5 attenuates efficiency of anti-PD-L1/PD-1 immunotherapy by inhibiting CD8(+) T cell-mediated anti-cancer immunity in bladder carcinoma. Adv. Sci..

