# Supplementary material for: A breast cancer classification and immune landscape analysis based on cancer stem-cell-related risk panel
Source: NPJ Precis Oncol. 2023 Dec 8;7:130. doi: 10.1038/s41698-023-00482-w (PMC10709318; doi:10.1038/s41698-023-00482-w)
Supplement: Supplementary file 1 — Supplementary Information [file 41698_2023_482_MOESM1_ESM.pdf]

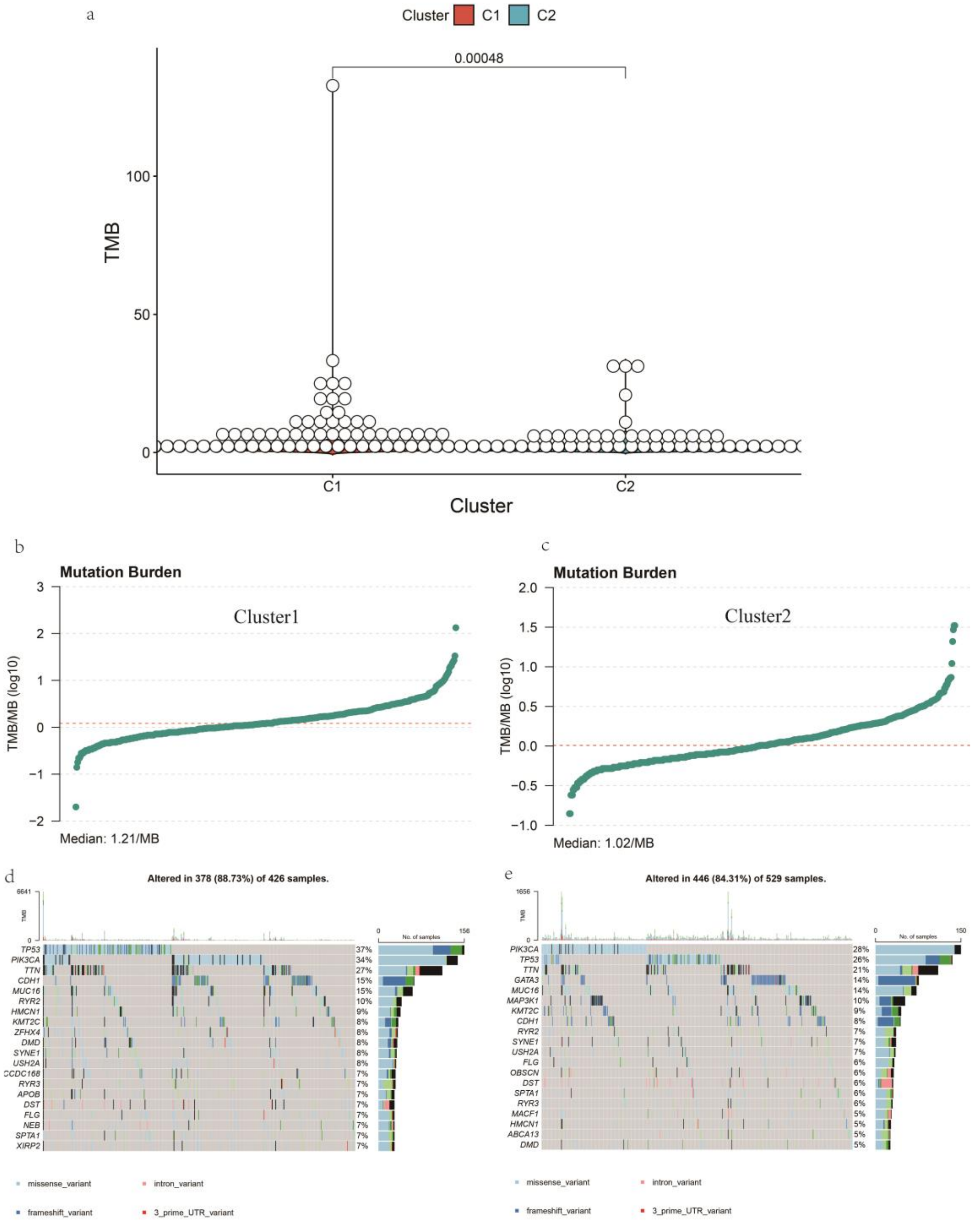


Supplementary Figure 1: Correlation analysis of tumor mutation TMB in Cluster1 and Cluster 2. (a-c) Differential analysis of TMB value in high- and low- risk groups. (d) Cascade of mutant genes in Cluster 1. (e) Cascade of mutant genes in Cluster 2.

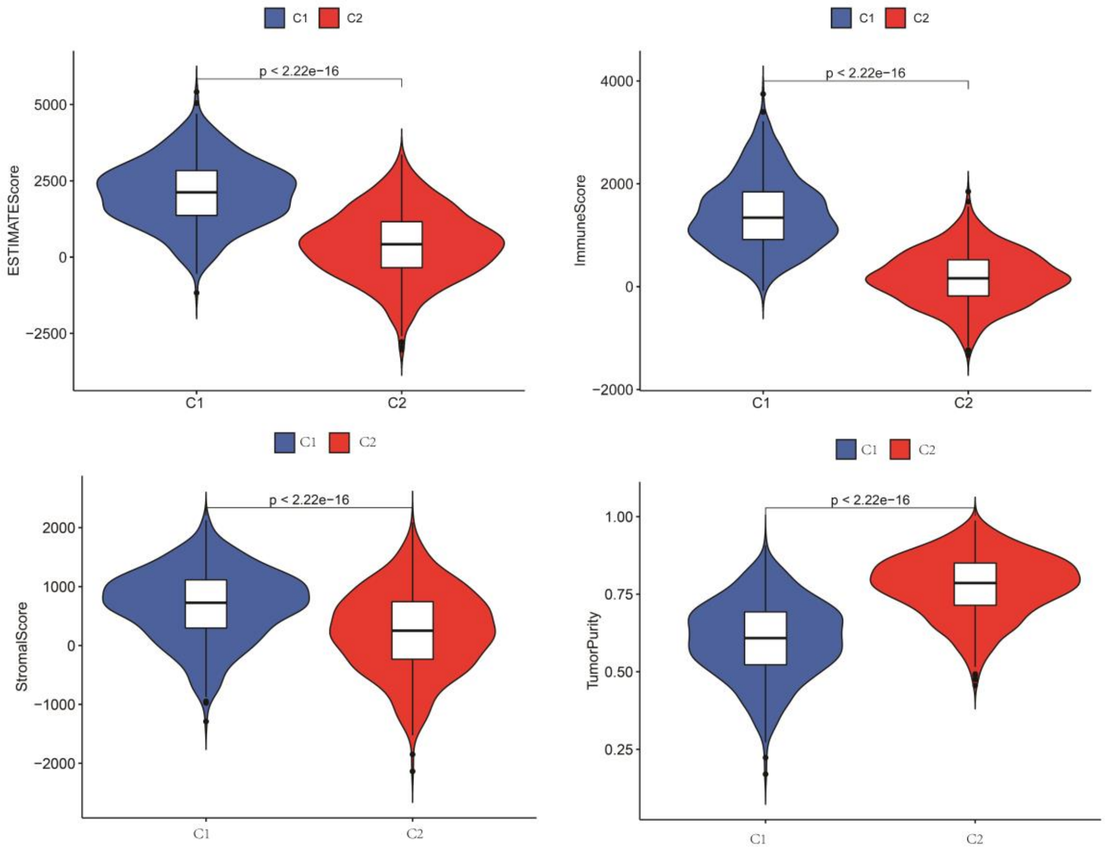


Supplementary Figure 2: Characterization of breast cancer stem cell-related subtype in tumor microenvironment.

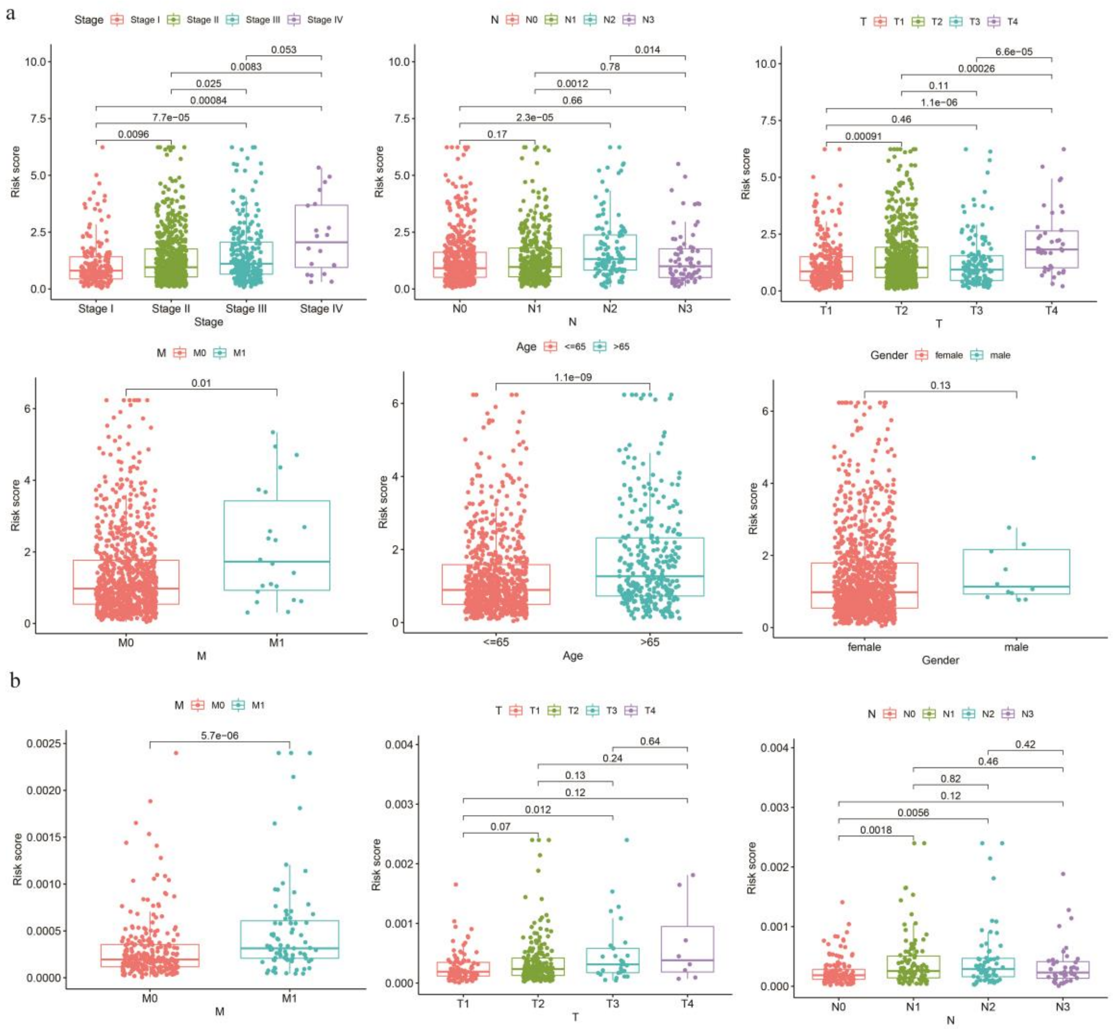


Supplementary Figure 3: Relationship between BCSCRS and tumor progression. (a) The relationship between BCSCRS and stage, age, gender, and TNM stage in the TCGA cohort. (b) The relationship between BCSCRS and TNM stage in the GSE20685 cohort.

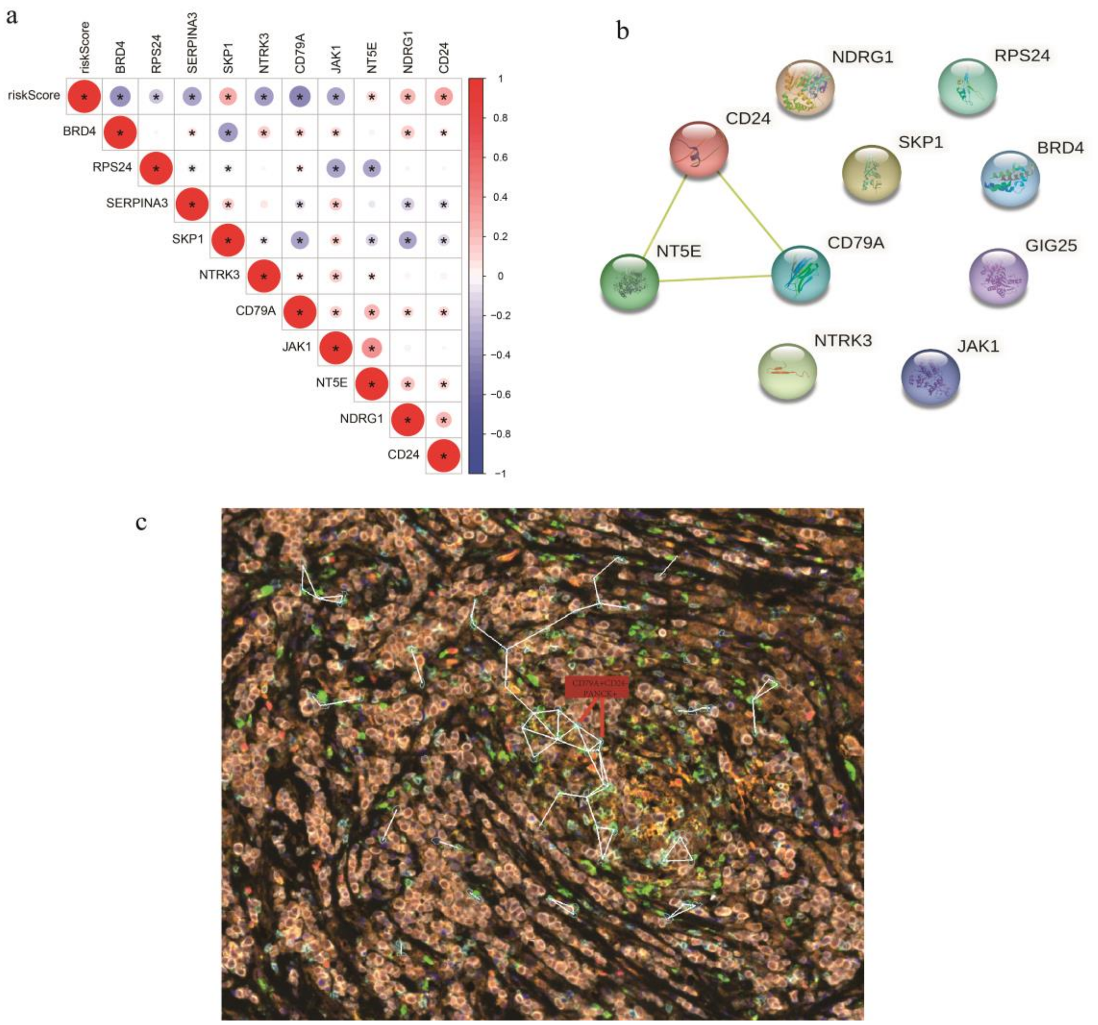


Supplementary Figure 4: The interaction of genes in risk panel. (a) Pearson correlation analysis between BCSCRS and each gene. (b) PPI regulatory network of genes in risk panel. (c) Identification map of a representative region of $CD79A^{+}CD24^{-}PANCK^{+}$-BCSCs subpopulation cell.
